# Complete Genome Sequence of a Shiga Toxin-Producing Escherichia coli O26:H11 Strain (Sequence Type 21) and Two Draft Genome Sequences of Listeria monocytogenes Strains (Clonal Complex 1 [CC1] and CC59) Isolated from Fresh Produce in Germany

**DOI:** 10.1128/MRA.00973-20

**Published:** 2020-12-03

**Authors:** Gregor Fiedler, Jan Kabisch, Erik Brinks, Sabrina Sprotte, Christina Boehnlein, Charles M. A. P. Franz

**Affiliations:** a Department of Microbiology and Biotechnology, Max Rubner-Institut (Federal Research Institute of Nutrition and Food), Kiel, Germany; University of Maryland School of Medicine

## Abstract

The complete genome sequence of a Shiga toxin-producing Escherichia coli (STEC) O26:H11 strain, MBT-5 (sequence type 21 [ST21], *stx*_1a_, *stx*_2a_, *eae*, *ehxA*), and two draft genome sequences of Listeria monocytogenes strains MBT-6 and MBT-7 belonging to the virulent sequence types 1 (ST1, clonal complex 1 [CC1]) and 59 (ST59, CC59), respectively, were determined. The strains were isolated in 2015 from ready-to-eat mixed greens in Germany.

## Abstract (precis)

The complete genome sequence of a Shiga toxin-producing Escherichia coli (STEC) O26:H11 strain, MBT-5 (sequence type 21 [ST21], *stx1a*, *stx2a*, *eae*, *ehxA*), and two draft genome sequences of Listeria monocytogenes strains MBT-6 and MBT-7 belonging to the virulent sequence types 1 (ST1, clonal complex 1 [CC1]) and 59 (ST59, CC59), respectively, were determined. The strains were isolated in 2015 from ready-to-eat mixed greens in Germany.

## ANNOUNCEMENT

Shiga toxin-producing Escherichia coli (STEC) serotype O26 can lead to life-threatening infections and is the second most common serotype (after O157) found in clinical and food samples in Europe (1, 2). Listeria monocytogenes primarily afflicts immunocompromised individuals and can cause listeriosis with a mortality rate of about 20% (3). Both pathogens may enter the food chain through raw animal and vegetable products (4). The strains in this study were isolated by enrichment and using selective agar from fresh salad greens as previously described in detail (5). In addition to the species and serotyping confirmation done previously (5), here, the genome sequence data were determined to aid in virulence assessment.

Cultures were grown overnight at 37°C in brain heart infusion (BHI) broth, and genomic DNA was extracted using the ZR fungal/bacterial DNA miniprep kit (Zymo Research, Freiburg, Germany) for *Listeria* and the Genomic Micro AX Bacteria+ kit (A&A Biotechnology, Gdynia, Poland) for E. coli. The AX Bacteria+ kit is suitable for getting high-molecular-weight DNA. DNA quantification and paired-end (MiSeq; Illumina) and long-read (MinION; Oxford Nanopore) sequencing were performed as previously described (6). For short-read sequencing, the Nextera XT library kit and the MiSeq reagent kit v3 were used, and for long-read sequencing, the PCR barcoding genomic DNA (SQK-LSK109) protocol vNBE_9065_v109_revR_14Aug2019 with a MinION MK1B instrument was used. Default parameters were used for all software. The raw reads were quality controlled using FastQC v0.11.7 (https://github.com/s-andrews/FastQC). Hybrid assembly of short and long sequence reads of STEC strain MBT-5, obtained from Illumina and MinION sequencing, respectively, was performed with Unicycler v0.4.8 (7). The assembly of L. monocytogenes strains MBT-6 and MBT-7 was done with SPAdes v3.10.0 (8), with Illumina-generated sequences only. Annotation was done on the assembled contigs using the NCBI Prokaryotic Genome Annotation Pipeline v4.11 (9).

The hybrid assembly of STEC strain MBT-5 generated a single circular chromosome of 5,705,580 bp, with 6 circular plasmids (pMBT-5.1, 92,578 bp; pMBT-5.2, 89,589 bp; pMBT-5.3, 34,196 bp; pMBT-5.4, 6,715 bp; pMBT-5.5, 4,742 bp; pMBT-5.6, 2,784 bp). The circular chromosome of MBT-5 was rotated to *dnaA* as the origin with Geneious v9.2. Plasmids were not rotated. Strain MBT-5 was identified as serotype O26:H11, sequence type 21 (ST21), and adhesin *fimH* type 440 using SerotypeFinder v2.0, MLST v2.0, and FimTyper v1.0, respectively (10–12). Virulence genes were found using VirulenceFinder v2.0 (Table 1) (13). Interestingly, plasmid pMBT-5.1 harbored the virulence genes *ehxA* (enterohemolysin), *espA* (extracellular serine protease), *toxB* (toxin B), and *katP* (catalase peroxidase), while all other virulence genes were located on the chromosome.

**TABLE 1:** Genome statistics of sequenced strains

Strain	Platform (no. of raw reads; avg length [bp])	Contig or no. of contigs	GC content (%)	Genome size (bp)	Contig type or *N*_50_ (bp)	Coverage (×)	No. of genes (CDS)^*a*^	ST (CC)^*b*^	Molecular Serotype^*c*^	Virulence genes determined by VirulenceFinder (total no.)^*d*^	GenBank accession no.	SRA accession no.
Escherichia coli MBT-5	Paired end using MiSeq (1,482,454; 251); MinION (127,117; 7,911)	Chromosome	50.7	5,705,580	Circular	260	5,790	ST25^*e*^	O26:H11	*astA*, *cif*, *eae*, *efa1*, *espB*, *espF*, *espJ*, *espP*, *fyuA*, *gad*, *iha*, *irp2*, *iss*, *iucC*, *iutA*, *lpfA*, *nleA*, *nleB*, *nleC*, *ompT stx*_1a_, *stx*_2a_, *terC*, *tir*, *tra* (chromosome); *ehxA*, *espA*, *katP*, *toxB* (plasmid 5.1) (29)	CP058682	SRR12207194 (paired end), SRR12207193 (MinION)
		Plasmid 5.1	47.8	92,578	Circular	281	93				CP058683	
		Plasmid 5.2	47.6	89,589	Circular	192	106				CP058684	
		Plasmid 5.3	39.7	34,196	Circular	386	36				CP058685	
		Plasmid 5.4	45.6	6,715	Circular	1,918	9				CP058686	
		Plasmid 5.5	41.2	4,742	Circular	1,151	4				CP058687	
		Plasmid 5.6	42.2	2,784	Circular	3,621	3				CP058688	
Listeria monocytogenes MBT-6	Paired end using MiSeq (669,662; 247)	21	37.93	2,879,121	515,847	44	2,874	ST1 (CC01)	4b	*agrA*, *flaA*, *flgC*, *flgE*, *gmar*, *lap*, *lapB*, *oatA*, *oppA*, *rli55*, *rli60*, *rsbv*, *bilE*, *bsh*, *chiA*, *clpB*, *clpc*, *clpe*, *clpp*, *codY*, *ctaP*, *ctsR*, *dal*, *degU*, *dltA*, *fbpA*, *fri*, *fur*, *hfq*, *hly*, *htrA*, *iap*, *inlA*, *inlB*, *inlC*, *inlH*, *inlJ*, *lgt*, *lhrC*, *lipA*, *lisR*, *lntA*, *lpeA*, *lplA1*, *lsp*, *mogR*, *mpl*, *mprf*, *murA*, *perR*, *pgdA*, *pgl*, *plcA*, *plcB*, *prfA*, *prsA2*, *pycA*, *recA*, *relA*, *secA2*, *sigB*, *sipX*, *sipZ*, *sod*, *srtA*, *srtB*, *stp*, *svpA*, *tcsA*, *tig*, *uhpT*, *virR* (72)	JACBGM000000000	SRR12207196 (paired end)
Listeria monocytogenes MBT-7	Paired end using MiSeq (616,456; 247)	24	37.83	2,975,327	482,161	45	2,985	ST59 (CC59)	1/2b	*agrA*, *ami*, *aut*, *flaA*, *flgC*, *flgE*, *gmar*, *lap*, *lapB*, *oatA*, *oppA*, *rli55*, *rli60*, *rsbv*, *biLE*, *bsh*, *chiA*, *clpB*, *clpc*, *clpe*, *clpp*, *codY*, *ctaP*, *ctsR*, *dal*, *degU*, *dltA*, *fbpA*, *fri*, *fur*, *gtcA*, *hfq*, *hly*, *htrA*, *iap*, *inlA*, *inlB*, *inlC*, *inlH*, *inlJ*, *inlk*, *lgt*, *lhrC*, *lipA*, *lisR*, *lntA*, *lpeA*, *lplA1*, *lsp*, *mogR*, *mpl*, *mprf*, *murA*, *perR*, *pgdA*, *pgl*, *plcA*, *plcB*, *prfA*, *prsA2*, *pycA*, *recA*, *relA*, *secA2*, *sigB*, *sipX*, *sipZ*, *sod*, *srtA*, *srtB*, *stp*, *svpA*, *tcsA*, *tig*, *uHpt*, *virR* (76)	JACBGL000000000	SRR12207195 (paired end)

a CDS, coding DNA sequences.

b See reference 11.

c See references 5 and 10.

d The relevant species was selected for (13).

e MLST scheme E. coli-1.

The assembly of L. monocytogenes strains MBT-6 and MBT-7 generated 21 and 24 contigs, respectively. No plasmid sequences could be determined. Genome characteristics are shown in Table 1. The molecular serotype and multilocus sequence type (MLST) were identified using MLST 2.0 and multiplex PCR as described earlier (5). Both L. monocytogenes strains showed genetic characteristics similar to those of previously reported clinical, food, and outbreak-associated populations (ST1 = clonal complex 1 [CC1] and ST59 = CC59) (14), indicating that virulent STEC and L. monocytogenes strains can be transmitted via fresh produce. However, these strains could be isolated only at low incidence in northern Germany in 2015 (5).

### Data availability.

The genome sequences have been deposited in GenBank under the BioProject number PRJNA643697. The sequences of STEC strain MBT-5 and L. monocytogenes strains MBT-6 and MBT-7 were deposited at DDBJ/ENA/GenBank under the accession numbers CP058682 (chromosome), CP058683 (plasmid 5.1), CP058684 (plasmid 5.2), CP058685 (plasmid 5.3), CP058686 (plasmid 5.4), CP058687 (plasmid 5.5), CP058688 (plasmid 5.6), JACBGM000000000, and JACBGL000000000. The raw sequencing data were also deposited at the SRA with the accession numbers shown in Table 1.
